# Oral squamous cell carcinoma arising from areas of Graft-versus-host disease: A systematic review

**DOI:** 10.4317/medoral.26133

**Published:** 2023-11-22

**Authors:** Marcelle Farias Silva Monteiro, Jeanne Gisele Rodrigues de Lemos, Flávia Sirotheau Corrêa Pontes, Alana Carla Silva da Silva, Misley Hellen Almeida Silva, Nathália Fernandes Silva, Lucas Lacerda de Souza, Daniel Cavallero Colares Uchôa, Hélder Antônio Rebelo Pontes

**Affiliations:** 1 ORCID: http://orcid.org/000-0003-2507-3823; Oral Diagnosis Department, Semiology and Oral Pathology Areas, Piracicaba Dental School, University of Campinas, Piracicaba, São Paulo, Brazil; 2 ORCID: https://orcid.org/0000-0002-6691-3764; Oral Surgery and Pathology Department, João de Barros Barreto University Hospital/Federal University of Pará, Belém, Pará, Brazil; 3 ORCID: https://orcid.org/0000-0002-1680-3102; Oral Surgery and Pathology Department, João de Barros Barreto University Hospital/Federal University of Pará, Belém, Pará, Brazil; 4 ORCID: https://orcid.org/0000-0002-0691-0502; Oral Surgery and Pathology Department, João de Barros Barreto University Hospital/Federal University of Pará, Belém, Pará, Brazil; 5 ORCID: https://orcid.org/0000-0003-1539-3399; Oral Surgery and Pathology Department, João de Barros Barreto University Hospital/Federal University of Pará, Belém, Pará, Brazil; 6 ORCID: https://orcid.org/0000-0003-4331-1864; Oral Surgery and Pathology Department, João de Barros Barreto University Hospital/Federal University of Pará, Belém, Pará, Brazil; 7 ORCID: https://orcid.org/0000-0002-9481-7796; Oral Diagnosis Department, Semiology and Oral Pathology Areas, Piracicaba Dental School, University of Campinas, Piracicaba, São Paulo, Brazil; 8 ORCID: https://orcid.org/0000-0002-3613-7071; Oral Surgery and Pathology Department, João de Barros Barreto University Hospital/Federal University of Pará, Belém, Pará, Brazil; 9 ORCID: https://orcid.org/0000-0002-7609-8804; Oral Diagnosis Department, Semiology and Oral Pathology Areas, Piracicaba Dental School, University of Campinas, Piracicaba, São Paulo, Brazil; 10 ORCID: https://orcid.org/0000-0002-7609-8804; Oral Surgery and Pathology Department, João de Barros Barreto University Hospital/Federal University of Pará, Belém, Pará, Brazil

## Abstract

**Background:**

Graft-versus-host disease (GVHD) is an immune system reaction that occurs in patients with a history of hematopoietic stem cell transplantation (HSCT), in which the grafted donor's cells attack those of the host. The objective of this systematic review was to present a study on oral squamous cell carcinoma (OSSC) that developed from GVHD areas in patients undergoing HSCT.

**Material and Methods:**

An electronic search was conducted in the databases PUBMED, WEB OF SCIENCE, SCOPUS, MEDLINE and SCIENCE DIRECT, according to PRISMA guidelines.

**Results:**

Of the 1582 results, 23 articles were included, resulting in 81 cases. The most common underlying disease for performing the transplant was Myeloid Leukemia (55.6%). The mean age was 39 years, with a predilection for males (64.2%). The tongue was the site of GVHD that most frequently underwent transformation to SCC (59.3%). The average time between transplantation and the development of GVHD was of approximately of 8 months, while the average period of development between transplantation and the development of OSCC was of approximately of 111 months. The most common treatment to GVHD was cyclosporine associated with corticosteroids.

**Conclusions:**

OSCCs arising from areas of GVHD present a different evolution from conventional oral carcinomas, since they affect younger patients, smoking and alcohol are not important etiological factors and finally because they present good prognosis, but further studies with larger number cases followed are needed to confirm our findings.

** Key words:**Graft vs host disease, squamous cell carcinoma of head and neck, transplantation, homologous.

## Introduction

Patients with hematological disorders currently have a higher chance of survival after the advent of hematopoietic stem cell transplantation (HSCT) (1-5). However, some patients treated with HSCT may develop certain short-, medium- and long-term complications. Among such disorders, the most significant is graft-versus-host disease (GVHD), as well as, neoplasias development from these GVHD sites (5-8).

GVHD is defined as a reaction that affects the patient's immune system, due the T lymphocytes acquired in the transplant attacking the host's tissues (2,5). This condition can negatively affect the quality of life of the transplanted patient, generating lesions in the skin, liver and oral mucosa (2,9).

GVHD occurs in 25% to 40% of patients who underwent HSCT after a long period of follow-up, while oral manifestations of GVHD occur in 35 to 60% of cases of acute hematological diseases that underwent transplantation and in 73 to 83% of chronic diseases (10,11). Oral squamous cell carcinomas (OSCC) are one of the most common solid tumors that develop in affected areas with GVHD, representing about 15% of these tumors (5,7,12,13).

The aim of this study was to perform a systematic review of the literature of cases of OSCC that developed in areas affected with GVHD.

## Material and Methods

This study followed the Preferred Report Items for Systematic reviews and Meta Analyses (PRISMA) guidelines (14). Moreover, the methods of this review were registered in the International Prospective Register of Systematic Review (PROSPERO) under protocol number CRD42022319457.

- Eligibility Criteria

This systematic review included cases of oral squamous cell carcinoma in transplant patients who developed in areas affected with graft versus host disease. The studies needed to have sufficient clinical and histological information to confirm the diagnosis. The histopathological aspects of squamous cell carcinoma were based on the latest World Health Organization Histological Classification of Head and Neck Tumors (15). Cohort studies, case-control studies, cross-sectional studies, randomized and clinical trials, case reports and case series written in English language were assessed.

Exclusion criteria were cases of SCC in the lip and skin region, cases of SCC without previous GVHD, cases in which the patient developed GVHD but did not develop SCC, and cases in which the histopathological diagnosis was carcinoma in situ. Review articles, immunohistochemical studies, histopathological studies, histomorphometry studies, genetic expression studies, cytological studies, cell proliferation/apoptosis studies, *in vitro* studies were excluded unless they presented cases with sufficient detail to confirm the diagnosis. Papers written in a language other than English were also excluded.

- Information source and Search strategies

An electronic search without time restrictions was undertaken up to October 2022 in the following databases: “PubMed”, “Science Direct”, “Web of Science”, “Scopus” and “MEDLINE”. The grey literature was also screened using Google Scholar database (Mountain View). The term used in the search strategies were “(oral squamous cell carcinoma OR OSCC) AND (graft vs host disease) AND (transplant)”. In a manual search were identified cases of OSCC in transplant patient with GVHD. Manual search was also conducted by crosschecking the reference list of the included studies in order to identify publications that might have been missed by the primary databases searches.

- Selection process

The titles and abstracts of all articles identified through the electronic search were read independently by three authors (MFSM, TFF and JGRL). Studies that appeared to meet the inclusion criteria or those in which there was not enough data in the title and abstract to make a clear decision was obtained as full paper copies for analyses and decision. Any disagreement was resolved by discussion between the authors. The clinicopathological and microscopic features described in the included studies was revised by two authors (HARP and FSCP) of the present study, who are experts in oral/maxillofacial pathology. All paper excluded at this stage of the screening process were documented along with the reasons for exclusion.

- Quality assessment

The risk of bias was carried out by using the Joanna Briggs Institute Critical Appraisal Checklist for case reports and case series (16). This tool classifies each study with a checklist comprising the study population, demographics, clinicopathological information, diagnosis, and follow-up, with available “yes”, “no” and “not available” answers. Thus, the studies were classified as “high”, “moderate” and “low” risk of bias. The analysis was performed by two authors (JGRL and ACSS) and a third author (HARP) was consulted for arbitration when disagreements occurred.

- Data extration

The available information from the eligible papers were collected and documented by two authors (NFS and MHAS) and then reviewed by a third author. A specially designed standard form in Microsoft Excel® software was used for the extraction of the following data, when available: author and year of publication, number of patients, age, sex, ethnicity, smoking, alcohol consumption, primary disease, OSCC site, histological confirmation, period between transplantation and GVHD, time of evolution from GVHD to OSCC, period between transplantation and development of OSCC, treatments before transplantation, drugs used in GVHD prophylaxis, treatments for GVHD, treatment for OSCC, survival time and recurrence.

- Synthesis Methods and Analysis

Qualitative and quantitative data were analyzed descriptively using Microsoft Excel® software. A narrative synthesis of the findings of the publications included was also carried out.

- Statistical analysis

The overall survival was evaluated by Kaplan-Meier curve method, whereas generated the general survival of the analyzed patients.

## Results

- PRISMA flowchart

**Figure 1:**
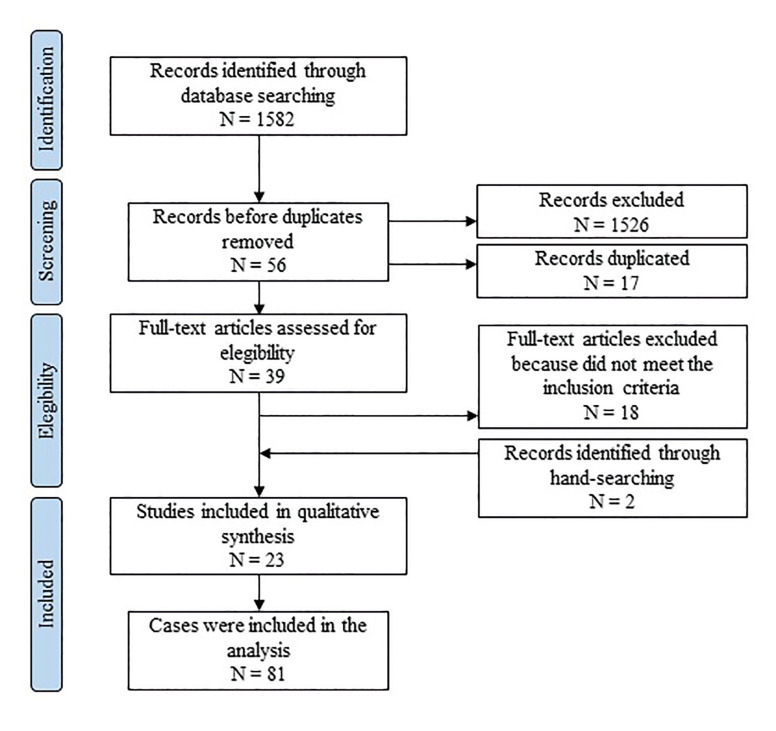
Screening process applied for the current study.

The study selection process is summarized in Fig. 1 (1-7,9,13,17-30). The search strategy identified 1,582 papers. The authors independently screened the titles and abstracts for those articles related to the study question. Of all papers, 1,526 were excluded for not being related to the topic, resulting in 56 records. Of these, 17 articles were cited in more than one database (duplicates).

The full-text reports of the 39 articles were evaluated and led to the exclusion of 18 articles, which did not present sufficient clinical and radiographic criteria to support the diagnosis, were confined to soft tissue or did not have bone tissue as their origin. Additional 2 records were included through hand- searching. Thus, a total of 23 publications were included in the review, comprising 81 cases.

- Description of the studies

In the present study, 23 studies published in the period from 1997 to 2021 were considered, which presented 81 cases of OSCC in patients treated with HSCT.

The clinicopathological data are summarized in Table 1.

**Table 1:**
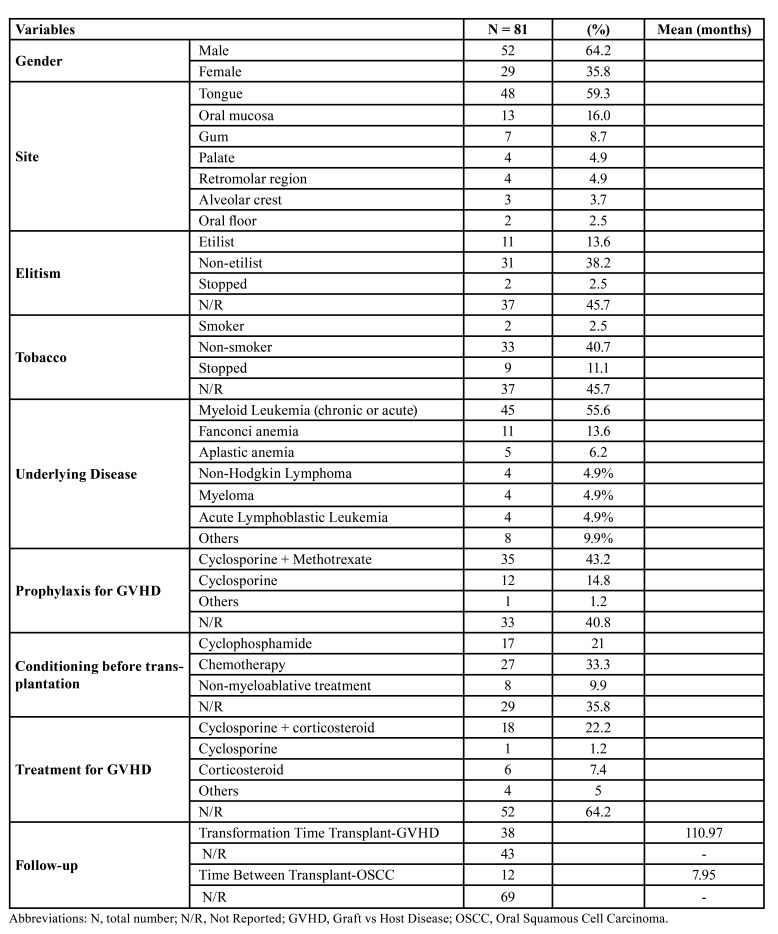
Demographic, clinical and radiographic features.

The reported cases showed a higher prevalence of carcinomas in males 64.2%, compared to females 35.8%, with a male-female ratio of 1.8:1. The mean age of 29 cases was 39 years, having a range between 12 and 53 years, 52 cases do not have this information. The most affected region was the tongue region, representing 59.3% of the informed regions. Tobacco consumption was reported in 2 cases (2.5%), in 33 cases (40.7%) the patient never smoked, 9 patients (11.1%) stopped smoking, and 37 cases (45.7%) did not report. Alcohol consumption was reported in 11 cases (13.6%) in 31 cases (38.2%) the patient never drank, 2 patients (2.5%) stopped drinking and 37 cases (45.7%) did not inform.

The primary diseases that led to HSTC were acute and chronic myeloid leukemia that affected 45 patients (55.6%), Fanconi anemia (11 cases; 13.6%), aplastic anemia (5 cases; 6.2%), anemia lymphoblastic acute (4 cases; 4.9%), non-Hodgkin lymphoma (4 cases; 4.9%), myeloma (4 cases; 4.9%), others (8 cases; 9.9%). The median time to development of GVHD after transplantation was 7.95 months (mean of 12 cases) and 69 cases do not report the time of development. The mean time for the diagnosis of OSCC after the development of GVHD was 110.97 months. The most used conditioning treatment was for immunosuppressive drug therapy with cyclophosphamide and chemotherapy, and for GVHD prophylaxis, the use of cyclosporine associated with methotrexate was most used. Considering GVHD control therapy, the main treatment was cyclosporine in association with prednisolone or azathioprine (Table 1).

- Quality assessment (Risk of bias)

**Figure 2:**
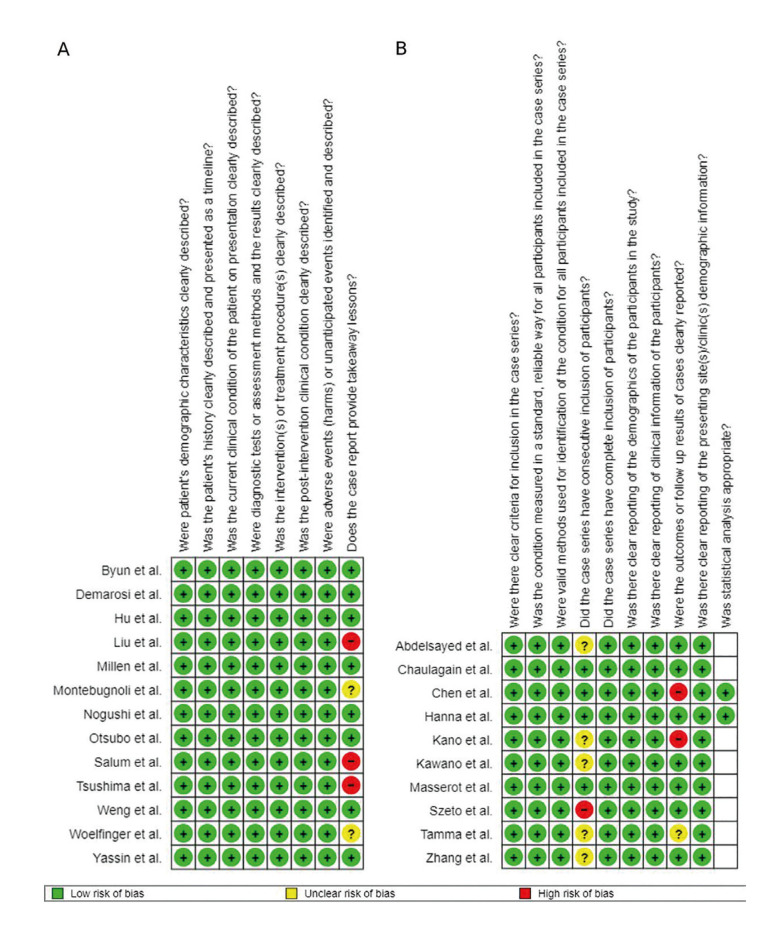
Risk of bias analysis chart for each article, according to the Joanna Briggs Institute Critical Appraisal Checklist. A) Summary of the case reports analyzed in the present study. B) Summary of the case series evaluate in the current research. The blank spaces refer to items not applicable to the included studies.

**Figure 3:**
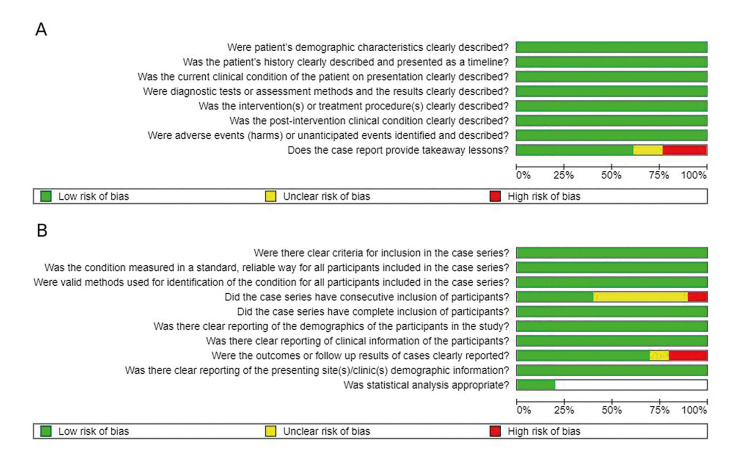
Chart of the summarized risk of bias analysis, according to the Joanna Briggs Institute Critical Appraisal Checklist. A) Overview of the case reports examined in each of the assessed study. B) Description of each case series investigated in the present research. The blank spaces refer to items not applicable to the included studies.

Following the Joana Briggs Institute risk of bias classification, overall low risk comprised 21 studies (12 case reports and 9 case series) and moderate overall risk of bias comprised 1 study, a case series (Fig. 2, Fig. 3).

- Survival analysis

**Figure 4:**
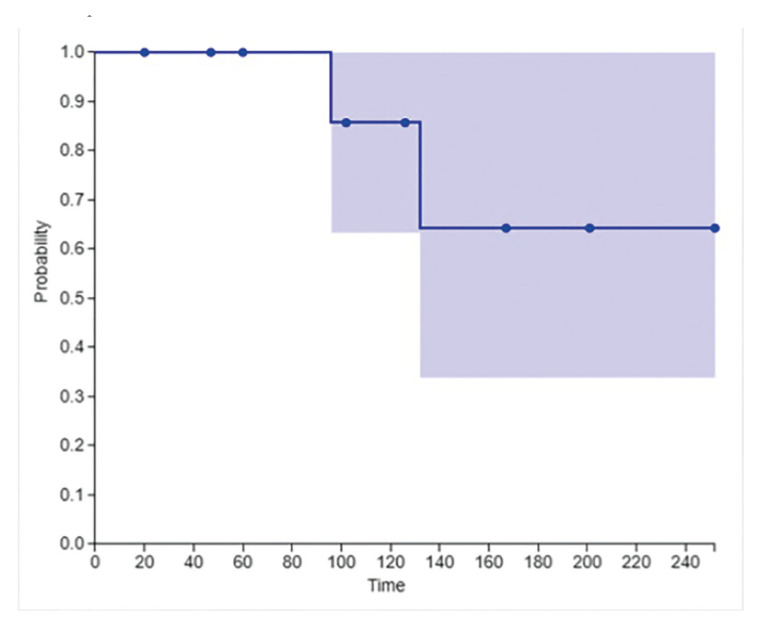
Kaplan-Meier graphic with an overall survival curve indicating that after 5 years, 100% of patients were alive.

Twelve patients had survival information available (Table 2). The overall survival rate of the analyzed sample was 100% and 86.4% 5 and 10 years of follow-up, respectively (Fig. 4).

**Table 2:**
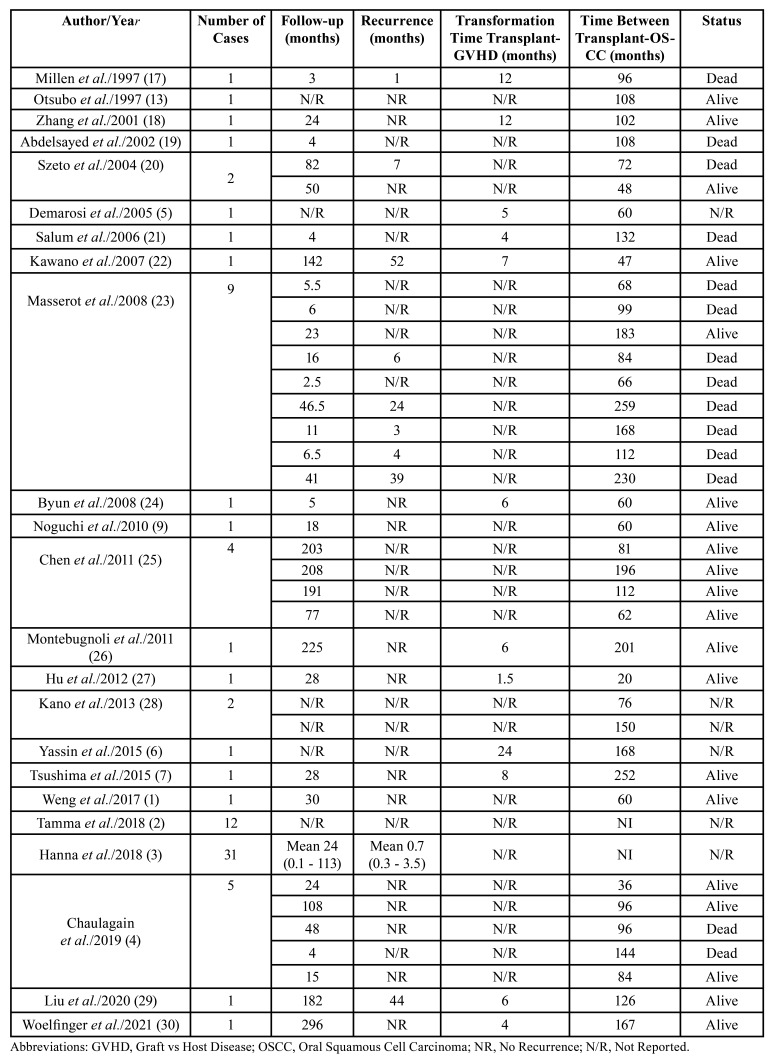
Follow-up time informations.

## Discussion

GVHD is a reaction of the immune system that occurs when cells from an immunocompetent patient are transplanted into an immunodeficient patient. The risk of developing malignancies secondary to HSCT is multifactorial and after transplantation the incidence of secondary malignancies increases over time (1,6,31-33). In this sense, the objective of this paper is to present a study on OSCC developed in areas of oral mucosa that had GVHD.

GVHD can present in an acute (aGVHD) or chronic (cGVHD) form, with the first manifesting up to 100 days after transplantation, and the latter occurring after this period (5). Skin, liver, lungs, gastrointestinal tract, eyes and oral cavity are the sites with the most clinical manifestations of cGVHD. Clinically, cGVHD oral lesions are similar to lichen planus, characterized by hyperkeratotic patches, striae, plaques, and papules. Erythema, atrophy, salivary gland hypofunction and oral pain may be noted. Severe ulcerations, often covered by a thin grayish-white to yellowish white pseudomembrane clot, may be present in more extensive and severe cases (1,5).

Patients with cGVHD resulting from HSCT are at increased risk of developing oral cancer, among these is OSCC (34). The pathogenesis for the development of OSCC after GVHD is not yet fully understood, however, many studies highlight some local factors that may influence the acquisition of the malignant phenotype, such as genomic instability that seems to be predisposed by chronic lesions immunologically mediated by T lymphocytes in oral GVHD, which, according to Khan *et al*., does not occur in other sites affected by cGVHD (8,32,34). The presence of inflammatory cells in areas affected by GVHD can provide a favorable environment for the evolution of carcinoma, due to the release of several molecules that participate in the initial stages of carcinogenesis (20,24). Other associated risk factors include pre- and post-transplant immunosuppressed status, immunosuppressive medication for GVHD prophylaxis, radiotherapy and chemotherapy for the treatment of the primary disease, immunoincompatibility between recipient and donor that leads to antigenic stimulation and interaction of these aspects in association with genetic predisposition (5,31,33). In this sense, oral squamous cell carcinomas arising from areas of graft-versus-host disease in the mouth showed a better prognosis than classic oral squamous cell carcinomas with a smoking-associated etiology. In our study, 100% of patients were alive after 5 years and 86.4% after 10 years. We hypothesize two theories for the best evolution of OSCC in patients who underwent HSCT. The first refers to a closer follow-up of the transplanted patient, allowing the identification of carcinoma at a less advanced stage. The second refers to a different pathogenesis between carcinoma that affects transplanted patients, such as the states of prolonged inflammation of the oral mucosa combined with the use of immunosuppressive drugs and cellular genetic mutations (32).

In our study, it was observed that cyclophosphamide was the most used drug in pre-transplant treatment. Cyclophosphamide is an alkylating agent widely used as an immunosuppressive drug and in the treatment of cancer, and although it has a selective ability to suppress regulatory T cells, cyclophosphamide can alter the number of myeloid-derived suppressor cells that contribute to tumor progression and angiogenesis (35,36). For GVHD prophylaxis, the use of cyclosporine in association with prednisolone or azathioprine was the regimen of choice in most studies. According to the literature, the use of cyclosporine with azathioprine and steroids is related to an increased risk of neoplasms, as well as treatments for GVHD with cyclosporine, azathioprine and thalidomine (5,24). In addition, when used alone as a treatment for GVHD, azathioprine is listed as an inducer of neoplasm (5,24).

In our study, the mean post-transplant GVHD transformation time was 239 days, in contrast with findings of Yassin *et al*. of 730 days (6). To the best of our knowledge, this is the first study reporting the time from GVHD transformation to OSCC after immunosuppression. The mean time found was 86 months for 23 patients, with the longest transformation time being 22 years reported by Janin *et al*. (37). The transformation time was shown to be varied, however, there were no important relationships with drugs or harmful habits that would indicate an influence on transformation times.

The classic etiological factors associated with OSCC in non-transplanted patients, smoking and alcohol do not seem to play an important role in oral carcinogenesis in areas of GVHD, since smokers were 2,5% of patients, while 40,7% did not smoke in the present work. Regarding alcohol consumption, it was observed that 38,2% of the patients did not consume alcoholic beverages and 13,2% used some type of alcohol. Although a high number of cases did not reported alcohol or tobacco use, the most cases occurred in young patients, there is not enough time for the harmful effects of these substances.

Because this study is a systematic review, it shows limitations. Some important datas could not be retrieved, such as the survival of most patients and more detailed information on tobacco and alcohol consumption.

In conclusion, OSCCs arising from areas of GVHD seem to be a different neoplasia from classical oral carcinomas, since they affect younger patients, as well as smoking and alcohol are not important etiological factors and finally because they present good prognosis, but further studies with larger number cases followed are needed to confirm our findings.
